# Radiomics Beyond Radiology: Literature Review on Prediction of Future Liver Remnant Volume and Function Before Hepatic Surgery

**DOI:** 10.3390/jcm14155326

**Published:** 2025-07-28

**Authors:** Fabrizio Urraro, Giulia Pacella, Nicoletta Giordano, Salvatore Spiezia, Giovanni Balestrucci, Corrado Caiazzo, Claudio Russo, Salvatore Cappabianca, Gianluca Costa

**Affiliations:** 1 Department of Life Sciences, Health and Health Professions, Link Campus University, 00165 Rome, Italy; f.urraro@unilink.it; 2 Department of Medicine and Health Sciences “V. Tiberio”, 86100 Campobasso, Italy; giulia_pacella@yahoo.it (G.P.); s.spiezia@studenti.unimol.it (S.S.); giovanni.balestrucci@studenti.unicampania.it (G.B.); corrado.caiazzo@unimol.it (C.C.); claudio.russo@unimol.it (C.R.); 3 Department of Precision Medicine, University of Campania “L. Vanvitelli”, 50138 Napoli, Italy; nicoletta.giordano@studenti.unicampania.it (N.G.); salvatore.cappabianca@unicampania.it (S.C.)

**Keywords:** radiomics, liver resection, segmentation, future liver remnant

## Abstract

**Background:** Post-hepatectomy liver failure (PHLF) is the most worrisome complication after a major hepatectomy and is the leading cause of postoperative mortality. The most important predictor of PHLF is the future liver remnant (FLR), the volume of the liver that will remain after the hepatectomy, representing a major concern for hepatobiliary surgeons, radiologists, and patients. Therefore, an accurate preoperative assessment of the FLR and the prediction of PHLF are crucial to minimize risks and enhance patient outcomes. Recent radiomics and deep learning models show potential in predicting PHLF and the FLR by integrating imaging and clinical data. However, most studies lack external validation and methodological homogeneity and rely on small, single-center cohorts. This review outlines current CT-based approaches for surgical risk stratification and key limitations hindering clinical translation. **Methods:** A literature analysis was performed on the PubMed Dataset. We reviewed original articles using the subsequent keywords: [(Artificial intelligence OR radiomics OR machine learning OR deep learning OR neural network OR texture analysis) AND liver resection AND CT]. **Results:** Of 153 pertinent papers found, we underlined papers about the prediction of PHLF and about the FLR. Models were built according to machine learning (ML) and deep learning (DL) automatic algorithms. **Conclusions:** Radiomics models seem reliable and applicable to clinical practice in the preoperative prediction of PHLF and the FLR in patients undergoing major liver surgery. Further studies are required to achieve larger validation cohorts.

## 1. Introduction

Liver resection is a curative treatment for both malignant and benign hepatic conditions [1,2]. The most frequent primary liver malignancies requiring resection are hepatocellular carcinoma (HCC) and intrahepatic cholangiocarcinoma (ICC) [3,4,5,6,7,8,9]. While MRI is commonly used for a differential diagnosis and treatment planning, contrast-enhanced computed tomography (CECT) remains essential for surgical planning, offering detailed anatomical and functional information [10,11,12,13,14,15,16]. A large volume hepatectomy, and in particular a hemi-hepatectomy, is often the preferred intervention for a curative treatment in various liver conditions [17,18], and it is strictly related to the future liver remnant (FLR) function and to the prediction of post-hepatectomy liver failure (PHLF) [19].

PHLF, defined as an inadequate liver function to meet metabolic demands, remains one of the most severe complications following liver resection, with a strong association to postoperative mortality [20,21]. Because its onset varies depending on the individual liver function, an accurate preoperative evaluation is key to minimizing risk [22,23]. Conventional assessments—such as a volumetric analysis, clinical scores, and liver function tests—lack sufficient predictive accuracy when used alone [24,25,26]. Emerging models, like the VIPP score, integrate multiple parameters to enhance prediction [27]. However, the variability in the liver regenerative capacity, influenced by the underlying disease and patient condition, continues to limit standardization [28,29].

Recent advances, especially regarding PET imaging, allow for a non-invasive regional liver function assessment, offering greater precision in patients with chronic liver disease or cirrhosis [30,31,32,33,34]. Accurate functional predictions can directly impact surgical decision-making and reduce complication rates [20,35]. The FLR remains the most reliable predictor of PHLF [36], but factors such as the patient frailty and surgical approach also contribute to outcomes [37,38,39,40].

Automated CT-based liver volumetry and 3D reconstructions are now commonly used to assess the FLR [41,42,43,44,45,46]. Deep learning (DL) algorithms have shown promise in segmenting Couinaud segments and liver lesions [47,48,49,50], though performance may be affected by liver attenuation changes due to steatosis or cirrhosis [51,52,53,54]. Additionally, the CT-based FLR estimation is influenced by the intravascular blood volume, introducing discrepancies between blood-filled (CT) and blood-free (surgical) volumetry [55,56,57,58,59,60]. Thus, a precise segmentation of hepatic and portal veins and a correction for the blood volume are essential for accurate assessments [61,62].

Radiologists play an increasingly central role in the multidisciplinary management of hepatobiliary (HPB) tumors, contributing to the diagnosis, surgical planning, and therapy selection [63,64,65,66]. Locoregional treatments are effective for early HCC and colorectal metastases and are expanding in use for ICC and pancreatic tumors [67,68,69,70,71,72].

Radiomics, by extracting quantitative features from CT and MRI, offers new opportunities to improve preoperative assessments [73,74,75,76]. Previous studies have demonstrated its utility in liver disease diagnosis, staging, and prognosis [77,78,79,80]. One limitation is the need for large datasets to train robust models [81], although physician-defined radiomic rules may help overcome this barrier [82]. The growing accessibility of artificial intelligence can support high-quality care in peripheral centers, including collaboration with transplant hubs [83,84,85].

The studies reviewed here explore how radiomics, particularly when integrated with preoperative CT imaging, can improve the prediction of PHLF and enhance surgical risk stratification, including the evaluation of the FLR.

## 2. Materials and Methods

This work follows a narrative review format, focusing on key studies relevant to the CT-based radiomics prediction of PHLF and the FLR. A literature analysis was performed on the PubMed Dataset (US National Library of Medicine http://www.ncbi.nlm.nih.gov/PubMed 11 October 2024). We reviewed original articles published up to July 2024, using a combination of the subsequent keywords: artificial intelligence, radiomics, machine learning, deep learning, neural network, liver resection, future liver remnant, and CT. The inclusion criteria comprised original research articles that applied radiomics, ML, or DL techniques to the CT-based evaluation of the FLR or the prediction of PHLF. We excluded case reports, editorials, letters to the editor, literature reviews, and preclinical or simulation-only studies. A preference was given to studies with external validation, clearly described segmentation strategies, and a sufficient patient cohort size. However, due to the narrative nature of this review, some degree of heterogeneity was tolerated. Although this is not a systematic review, the quality of the included studies was discussed with reference to the CLEAR checklist (Checklist for Artificial Intelligence in Medical Imaging, 2023) [1], and although no formal scoring was applied, there was a qualitative consideration of key elements from the CLEAR checklist—such as the external validation, feature reproducibility, data availability, and clinical applicability.

## 3. Results

A total of seven eligible studies were included and categorized based on the target prediction (FLR or PHLF), model type (ML, DL, nomogram), and validation strategy (internal, external, not clearly defined). Table 1 provides a summary of the included studies.

According to the CLEAR checklist recommendations [1], a qualitative assessment of the methodological transparency of these studies was conducted, especially regarding the presence of an external validation, the clarity of segmentation protocols, the reproducibility of the radiomic feature extraction, and the interpretability of the models. Some studies, like those by Xie et al. [87,88], demonstrated methodological rigor, clear segmentation procedures, and a robust external validation. Others, however, lacking in some elements, may have limited results in terms of reproducibility and potential clinical applications.

## 4. Discussion

Although we did not apply a formal scoring system, like TRIPOD or PROBAST, many of the studies included in this review showed some important methodological limitations. Several models were developed using small, single-center datasets, which increases the risk of overfitting and makes the results harder to apply to other patient populations. In most cases, the segmentation methods were not blinded or tested for reproducibility, which could lead to a wide variability depending on who performs the analysis. Only a few studies, such as those by Xie et al. [87,88] included an external validation with independent data, which is essential for confirming that the model works outside the original setting. The reporting of key aspects like the model inputs, how the validation was performed, and whether the models were calibrated was often incomplete. Finally, none of the studies made their data or code publicly available, which limits the possibility for others to reproduce or improve these models in the future.

### 4.1. Prediction of Post-Hepatectomy Liver Failure

Post-hepatectomy liver failure (PHLF) is the most worrisome complication after a major hepatectomy and is the leading cause of postoperative mortality, with an incidence after liver resection that ranges from 0.7 to 39.6% [91,92,93]. Moreover, PHLF is the major cause of prolonged hospitalization, increased costs, and poor long-term outcomes in patients undergoing a hepatectomy. The 2011 International Study Group of Liver Surgery (ISGLS) grading scale for PHLF reports that the grades A, B, and C are associated with mortality rates of 0, 12, and 54%, respectively [94]. Several recent studies emphasize the multifactorial risks related to the development of postoperative liver failure, including an underlying liver disease, such as cirrhosis or chronic hepatitis. These chronic conditions influence the liver function and regeneration after surgery [95]. This aspect underlines the importance of considering liver disease and quantifying the liver function in PHLF prediction models [96,97]. The latest advancements in hepato-biliary surgery have underscored the critical need for the precise identification of the localization of the disease and for a prediction of the remnant liver function before a major hepatectomy, to prevent PHLF as a significant cause of morbidity and mortality [98]. Numerous predictors have been explored to develop a tool that accurately assesses the preoperative risk, yet a consensus has not been reached, partly due to the variability in the liver function among patients [25,99,100]. Additionally, a tool for an accurate prediction of the risk for PHLF preoperatively assists with patient selection and earlier interventions to prevent PHLF. Nowadays, the reported predictors of PHLF are the subsequent: the indocyanine green (ICG) clearance [101,102], “50–50 Criteria”, model for end-stage liver disease (MELD) system [103], Child–Pugh grade, and future liver remnant (FLR) volume [104,105]. Furthermore, multivariable models have been created to merge the predictive power of these markers and create a more reliable model to predict the risk of PHLF. However, there is still a lack of a standardized tool for clinical practice. 

The “50–50 criteria” consist of the presence of both a PT < 50% and an SB > 50 μ mol/L in the fifth postoperative day [106], but if the MELD is incorporated, the predictive power of the score is increased in a population of patients undergoing surgery [107,108,109]. 

In the same way, serological biomarkers such as bilirubin, albumin, INR, and the platelet count, integrated with the MELD score, have long been used in liver function assessments. Also, emerging biomarkers such as the indocyanine green clearance, hyaluronic acid, and PT activity have been included in predicting PHLF [110,111]. These innovative biomarkers, etiological data, and MELD scores have been studied to build a DL model in the study by Kang et al. [20]. The authors aim to provide a more comprehensive and clinically relevant tool for the preoperative assessment of the PHLF risk, emphasizing the need for a multifactorial risk stratification in liver resection procedures, thereby enhancing the precision and personalization of surgical planning [112]. Their DL model—based only on the liver-specific loss function, the “weighted liver loss” function—was built from the portal phase of CT scans of 52 patients and was compared to existing ML and DL techniques [113].

A primary reason for utilizing this model design is its considerable depth and complexity, which allows the model to find intricate patterns and interactions among the 23 medical features. According to the previous literature, data collection included other parameters of the liver function definition, such as liver-related laboratory indicators [114], the model for end-stage liver disease (MELD) [20], and the aminotransferase-to-platelet ratio index (APRI), which are used in various clinical settings to evaluate liver damage and predict outcomes in liver diseases [115]. PHLF was defined on the “50–50 criteria”, which means the concomitant presence of a PT < 50% and an SB > 50 μ mol/L on postoperative day 5. The key contribution of Kang et al. [20] is the introduction of the tailored “weighted liver loss” function, designed to accurately predict the volume of the liver tissue to be resected during surgery. This innovative function addresses prediction errors in a nuanced manner by differentiating between the clinical implications of overestimations and underestimations—assigning higher penalties to under-predictions due to their greater risk to patient safety—while traditional loss functions treat all deviations equally. By combining weighted and unweighted squared errors, it balances clinical safety with the error magnitude, and its flexible design, featuring adjustable parameters for under-penalties and over-penalties, allows for customizations to meet different clinical needs and scenarios. [26]

The model achieved a significantly higher Percentage of Successful Resection Volume (*p* = 68.88%) compared to traditional models based on linear regression (*p* = 48.22%) and Support Vector Regression (*p* = 45.77%). It also had the lowest Mean Absolute Error for under-predictions (23.72%) and a significantly lower Mean Absolute Error for over-predictions compared to models such as Polynomial Regression and Random Forest, indicating a more precise estimation of safe resection limits. These findings highlight that the bespoke model, with its specialized loss function, provides a more accurate and reliable method for predicting safe liver resection volumes. This approach minimizes the risk of PHLF, ensuring that the surgical removal of the liver tissue is both safe and effective, ultimately aligning with clinical priorities and addressing critical needs in the preoperative planning for liver surgery. 

DL with convolutional neural networks (CNNs) has been proven to have clinical significance in various medical image interpretation tasks. Recent research showed that a DL model, based on medical data, could be used for the preoperative prediction of severe liver failure after a hemi-hepatectomy in patients with hepatocellular carcinoma. A Fully Connected Feedforward Neural Network (FCNN) produced the most accurate predictions among standard models, reinforcing the effectiveness of DL in capturing complex patterns in medical data. In this context, the loss function tailored approach of Kang et al. [20] surpassed all other models, achieving a 68.88% success rate in predicting resection volumes and significantly lower Mean Absolute Errors for both under- and over-predictions. This demonstrates that specialized algorithms can outperform generalized methods in medical applications, setting a new benchmark in the field of liver resection volume prediction [116,117,118]. In the same field of DL models’ evaluation as PHLF predictors, the aim of Xu et al. [89] was to build and validate a DL model of a presurgical PHLF prediction using the preoperative multiphasic CECT (contrast-enhanced computed tomography), including the non-contrast, arterial phase, and venous phase, of the 265 enrolled patients with various liver conditions. The DL model was evaluated using a 5-fold cross-validation to predict PHLF. The overall accuracy of the predictions was 84.15%, with an AUC of 0.7927. Notably, the model achieved a higher accuracy in left hemi-hepatectomy cases (89.41%) and showed a good predictive performance across different liver conditions; more in detail, the model achieved a 77.47% accuracy for the liver mass, a 78.33% accuracy for liver cirrhosis, and an 80.46% accuracy for viral hepatitis. This demonstrates the model’s potential effectiveness in assisting in the surgical planning and risk assessment for liver resections. The key finding of this deep learning model includes that the model helps in selecting patients for hemi-hepatectomies, enabling better preoperative preparations: high-risk patients identified by the model could be considered for alternative treatments like a portal vein embolization (PVE), ALPPS, radiofrequency ablation (RFA), or transcatheter arterial chemoembolization (TACE) [119,120]. Despite the promising results, the study was affected by limitations, including the lack of a multimodal algorithm in the medical evaluation of surgical liver patients, as seen in other studies. So, the model shows significant potential, but further research is necessary to enhance its performance and ensure its reliability across different clinical settings. The challenge of this was based on the comprehension that a multivariable preoperative approach could improve the predictive goal in the evaluation of PHLF better than the prediction of single methods, especially in patients undergoing extensive liver resections due to large tumor diameters and major vascular invasion. In these cases, the morbidity was 10.9–43.6% and the mortality was 4.2–18.1% [121]. Of course, the incidence of PHLF increases with the percentage of the liver that has been resected [122]. Therefore, establishing an individualized prediction model for PHLF in patients with huge HCC is critical. 

The study of Xiang et al. [24] aimed to establish a radiomics-based nomogram, analyzing livers affected by chronic liver disease, for predicting severe (grade B or C) PHLF in patients with huge (≥10 cm) hepatocellular carcinoma (HCC). A total of 186 patients with huge HCC that underwent curative hepatic resections were included and divided into a training dataset (131 patients) and a test dataset (55 patients). The LASSO method was used to identify a radiomics signature from the training dataset, with the aim of predicting grade B or C PHLF. The radiomics signature comprised nine features. For the radiomics nomogram creation, a multivariable logistic regression model was developed, which incorporated the radiomics signature and other clinical predictors, with the result of a radiomics nomogram designed to predict severe PHLF. According to a decision tree analysis, the model stratified patients into different risk categories for severe PHLF. For this aim, the study successfully developed a radiomics signature and a radiomics nomogram that could predict severe PHLF with a high accuracy (AUC for training dataset: 0.766; AUC for test dataset: 0.745). The nomogram, which integrates radiomics features and clinical predictors such as the extent of the resection and the MELD score, showed an excellent discrimination ability (AUC for training dataset: 0.842; AUC for test dataset: 0.863). The decision tree analysis stratified patients into low- (radiomics score < −0.247 and MELD score < 10), intermediate- (radiomics score < −0.247 but MELD score ≥ 10), and high-risk categories (high-risk patients: radiomics score ≥ −0.247 and underwent extended resections), facilitating a personalized risk assessment and potentially guiding clinical decision-making for patients undergoing hepatic resection.

Other recent studies have explored radiomic features in the prediction of the PHLF occurrence, [123] reporting that the PHLF–liver parenchyma exhibits a more heterogeneous appearance as compared to normal patients. ML can improve the selection of significant features as radiomics signatures [124]. At the end of this valuation, the hypothesis emerged that a radiomics nomogram could play an important role in predicting severe PHLF in patients who need major hepatectomies or with larger tumors or to achieve R0 resections, to improve a precision medicine-based approach. Likewise, according to Cai et al. [90], a PHLF predictive biomarker integrating liver and tumor features could be derived from preoperative CT data and introduced into a radiomics score (Rad-score), developed using logistic regression with LASSO regularization and combining multiple prognostic factors, to create a radiomics-based nomogram incorporating the Rad-score, MELD score, and performance status (PS). This study by Cai et al. [90] included 112 HCC patients (divide into 2 groups: 80 in the training cohort and 32 in the validation cohort), with an extra 13 patients in a pilot prospective analysis. From portal-phase CT images, 713 radiomics features were extracted to develop a radiomics score (Rad-score) using logistic regression. A nomogram incorporating the Rad-score and other risk factors was built using a multivariate logistic regression model. However, the Rad-score alone did not demonstrate a better performance than the Child–Pugh (CP) score, model for end-stage liver disease (MELD), and albumin–bilirubin (ALBI). The radiomic model achieved an AUC of 0.822 in the training cohort and an AUC of 0.762 in the validation cohort. Conversely, the Combined Nomogram Performance, incorporating the Rad-score, MELD, and performance status, showed an improved discrimination and was significantly superior to the other methods alone (Training cohort: AUC of 0.864 (95% CI, 0.786–0.942). Validation cohort: AUC of 0.896 (95% CI, 0.774–1.000)). The pilot prospective analysis shows that the radiomics nomogram predicted PHLF with an AUC of 0.833 (95% CI, 0.591–1.000).

The study by Cai et al. [90] successfully developed a radiomics score and a comprehensive nomogram that predicted PHLF more accurately than traditional methods. The nomogram, combining the Rad-score, MELD, and performance status, demonstrated a superior discrimination and clinical utility, suggesting its potential for improving the preoperative planning and risk assessment in HCC patients undergoing a hepatectomy. 

Unfortunately, further validation and enhancement are still needed; multicenter studies and the inclusion of additional clinical data are necessary for better reliability and adaptability. In the end, the accurate prediction of the liver resection volume is crucial for enhancing surgical planning and reducing postoperative complications. The integration of AI highlights its potential in medical decision-making due to its capability to analyze complex data and to provide precise predictions, thereby improving preoperative planning for major hepatectomy and producing lower rates of postoperative complications [125,126,127].

**Figure 1:**
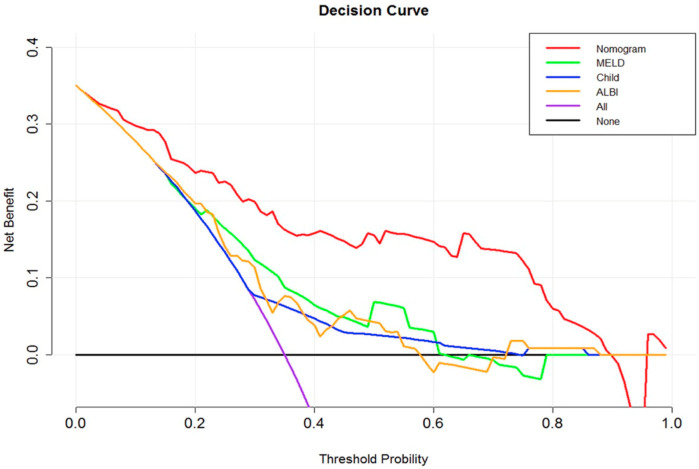
Adapted from Cai et al. through Elsevier license number 6070981060477 [90]. Decision curve analysis of the radiomics nomogram comparing the net clinical benefit of the radiomics nomogram versus conventional models (MELD, Child–Pugh, and ALBI) and default strategies (treating all or none). The nomogram outperforms traditional scores across a wide range of threshold probabilities, indicating its superior clinical utility in the preoperative prediction of PHLF.

In summary, recent developments in radiomics and deep learning have shown promising results for predicting PHLF. Several models, including those by Kang et al. [20], Xu et al. [95], Xiang et al. [24], and Cai et al. [90], have combined clinical and imaging data to improve preoperative planning as shown in Figure 1. However, there are still important limitations. Many studies use different definitions of PHLF, include very different types of patients, and follow different imaging protocols. Also, most models have not been tested in external or multicenter datasets and often rely on features that are specific to a single hospital or research group. Because of this, the results may not effectively apply to other settings. While the early results are encouraging, more research with larger and more diverse patient groups is needed before these tools can be used in routine clinical practice.

### 4.2. Prediction and Functional Evaluation of Future Liver Remnant

As the incidence of PHLF reaches 58% in the case of a major hepatectomy, the preoperative evaluation of the functional future liver remnant (FLR) appears to be critical and necessary to avoid PHLF [128]. Currently, in clinical practice, the estimation of the functional liver volumetry is based on blood tests such as the ALB, AST, TBIL, and indocyanine green retention at a 15 min retention rate (ICG-R15) [129]. Radiologists can estimate the liver volumetry, segmenting 3D reconstructions of the entire liver parenchyma excluding vascular structures. However, this can be useful to describe the percentage of disease but not to quantify the liver function which belongs to the actual scoring systems: the MELD score, the Child–Turcotte–Pugh (CTP) score, and the albumin–bilirubin (ALBI) grade. By comparing these indexes, the ICG-R15 shows more advantages, such as the earlier and more accurate detection of abnormalities and their proven relation to liver failure and morbidity after a hepatectomy [130]. In detail, ICG-R15 values of the patient in different intervals (threshold: 10%, 20%, and 30%) can affect and guide the selection of surgical treatment methods.

As reported by the Makuchi criteria of safe hepatic resections, if the ICG-R15 is <10%, this may allow a tri-segmentectomy and bi-segmentectomy; for values between <10% and <20%, a left lobectomy or right mono-segmentectomy of the liver can be performed; for values between 20% and 30% a sub-segmentectomy of the Couinaud of the liver can be performed; and for an ICG-R15 ≥ 30%, it is necessary to limit the resection or enucleation of the liver for transplantation [131]. The ICG clearance test seems to be considered as an optimal standard tool to assess the preoperative FLR [132]. As the volumetry of the FLR does not exactly reflect the function of the FLR, especially in pathological livers, this method, based on Couinaud segment asportation, can have several limitations [133]. Beyond anatomical details, radiomics has been proposed as an innovative tool in liver oncological disease [134] and also in measuring the hepatic function in HCC patients, providing more significant clinical information than an overall assessment [135]. In this field, recent studies report that ML approaches can relate quantitative imaging features and clinical manifestations [136]. Moreover, considering that the volumetry on CT images is starting to be more accessible and reliable, thanks to the several models of automatic segmentation which appears to be fast and accurate, ensuring the CE-CT as the gold standard in the evaluation of the FLR and radiomics models [137,138]. Radiomics models derived from CECT imaging have shown promising results in assessing the FLR, especially when compared to established functional evaluation tools such as the ICG-R15, which remains a clinically validated and widely used test for evaluating the liver functional reserve before a hepatectomy.

Among the ML classifiers developed to analyze CECT–radiomic features to predict the FLR, the XGBoost model demonstrated a superior predictive performance by effectively combining multiple weaker models into a strong ensemble prediction, making it one of the most popular algorithms for classification and regression tasks in medical imaging [86,139]. Specifically, the study by Zhu et al. [86] explored the capability of ML models, built on radiomic features from CECT images, to evaluate the FLR function in HCC patients undergoing liver resection. They used the ICG-R15 as the reference standard to categorize the liver functional reserve, providing an objective clinical benchmark for their predictive models. This approach highlights the potential of integrating imaging biomarkers with clinical functional tests to improve preoperative assessments and surgical planning. The preoperative CT images of 190 HCC patients were retrospectively enrolled and randomly classified into a training and a test dataset. Then, 107 radiomics features from portal-phase CT images were extracted, and the features related to the ICG-R15, which was classified into 10%, 20%, and 30%, were selected. Five ML classifiers were used for the ML model investigation. The XGBoost classifier exhibited a superior performance at an ICG-R15 threshold of 10%, achieving an AUC of 0.822 and an accuracy of 0.842. At an ICG-R15 threshold of 20%, the SVM classifier outperformed others with an AUC of 0.860 and an accuracy of 0.842. At an ICG-R15 threshold of 30%, the XGBoost classifier once again demonstrated the highest efficacy, with an AUC of 0.938 and an accuracy of 0.965. These results highlight the differential efficacy of the classifiers across various levels of hepatic functionality, as indicated by the ICG-R15 values, underscoring the importance of selecting appropriate models based on specific clinical conditions. Xie et al. aimed to develop a deep learning model for the automatic assessment of both the FLR and of the liver Couinaud segmentation; it was tested in an external validation cohort to evaluate the feasibility of a major hepatectomy considering the risk of PHLF but also producing a 3D visualization for surgeons [87,88].

Several other studies have explored or developed DL algorithms for the evaluation of the liver volumetry [140] or for the automated segmentation of hepatic veins and portal veins [141], which can potentially be used in preoperative FLR assessments prior to a major hepatectomy, but no one analyzed the volumetric difference between the blood-free and blood-filled FLR and rarely considered the external validation via the use of various pathologic livers, like in this case.

Indeed, the segmentation of hepatic vessels poses a major challenge in presurgical planning, particularly in livers affected by cirrhosis or the vascular invasion from hepatic tumors, where veins may appear smaller and blurred. Xie et al. noted that the larger the volume of blood vessels, the greater the discrepancy between the measured FLR and the real FLR [87]. This issue is because the preoperative CT volumetry of FLR can under- or overestimate the real FLR due to blood-filling effects, whereas the intraoperative volumetry is examined in a blood-free setting. To address this, a DL model was validated for the automatic segmentation of hepatic and portal veins and was applied for a presurgical FLR% assessment in both blood-filled and blood-free contexts. In the work of Xie et al., the “3D U-Net” DL model was built on CECT porto-venous phase scans from 170 patients [87]. Following the manual annotation of Couinaud segments by a radiologist, their “3D U-Net DL” model was trained and tested on datasets encompassing various liver conditions as well as candidates for a major hepatectomy. The segmentation accuracy was evaluated using the dice similarity coefficient (DSC), and the quantitative volumetry for assessing the resectability was compared between manual and automated methods. Both approaches reached highly similar results in evaluating the FLR and FLR% across hepatic segments I–VIII, with DSC values ranging from 0.93 to 0.95. In the test datasets, the mean automated FLR and FLR% were 493.51 ± 284.77 mL and 38.53 ± 19.38%, respectively, compared to 500.92 ± 284.38 mL and 38.35 ± 19.14% for manual assessments. A statistical analysis confirmed no significant differences between the two methods in the FLR (*p* = 0.50; U = 185,545), the FLR% (*p* = 0.82; U = 188,337), or the indications for a major hepatectomy (McNemar test statistic 0.00; *p* > 0.99). These findings suggest that automated segmentation can serve as an effective alternative to manual segmentation in the preoperative evaluation of liver resection candidates, with the potential to streamline and enhance the accuracy of presurgical planning. The key contribution of this study is the demonstration that preoperative FLR% assessments and resection predictions are fully comparable between these settings and across various types of hepatectomies and liver conditions. Moreover, when compared to human performance, the DL models achieved similar results in predicting resection outcomes in an external validation dataset [87]. Overall, this study underscores the feasibility of automated preoperative assessments of the blood-free FLR and FLR%, providing a reliable tool for clinical decision-making in liver surgery.

A further study by Xie et al. [88] developed and validated a deep learning (DL) model for the automated segmentation of hepatic and portal veins and the assessment of the future liver remnant (FLR) in a blood-free setting on preoperative CT scans before a major hepatectomy. The assumption was that the preoperative CT volumetry usually under or overestimates real volumes mostly because of the blood volume contained in large hepatic vessels, while intraoperative measurements are blood-free [142]. Since the blood pool comprises more than 9% of the whole liver volume, and considering that the FLR ranges from 20 to 40%, it should be considered to limit the error of the preoperative CT volumetry [36]. The model, based on a 3D U-Net network, was trained on a dataset of 170 patients and tested on an external validation set of 178 patients with various liver conditions. The segmentation accuracy was evaluated using the dice similarity coefficient (DSC) and Volumetric Similarity (VS), with mean DSC values of 0.66 for hepatic veins and 0.67 for portal veins. No significant differences were found between the FLR and FLR% in blood-filled and blood-free settings, using either manual or automated methods (*p* = 0.67 and 0.66 for FLR, *p* = 0.59 and 0.99 for FLR%). Additionally, the model demonstrated a performance comparable to that of radiologists in predicting the liver resectability. This study highlights the potential of DL to enhance preoperative planning by providing an automated and accurate FLR assessment, regardless of the presence of blood in hepatic vessels, which could help reduce the risk of post-hepatectomy liver failure (PHLF).

In summary, an FLR evaluation is essential in a presurgical setting because it helps reduce the PHLF risk. Traditional tools, like the ICG-R15; liver volumetry; and clinical scores such as the MELD or ALBI, are widely used but with some limitations, especially in patients with liver disease. Recent ML and DL studies, such as models developed by Zhu et al. [86] and Xie et al. [87,88], have improved the accuracy of FLR assessments and offer helpful tools for preoperative planning. However, these models often lack external validation, and many are based on single-center data or limited patient groups. Also, some important factors like vascular changes or liver inflammation are not fully considered. While AI-based models show strong potential, more research—especially multicenter studies—is needed to confirm their reliability and make them usable in real-world clinical practice.

### 4.3. Remaining Gray Areas and Future Perspectives

Liver tumors—including hepatocellular carcinoma (HCC), intrahepatic cholangiocarcinoma (ICC), benign lesions such as adenomas or hemangiomas, metastatic liver disease, liver trauma, biliary diseases such as cholangitis, and refractory or complicated liver abscesses—often require liver surgery [36,142,143,144]. A partial hepatectomy remains the optimal treatment for HCC, despite many patients presenting at advanced stages due to insidious symptoms [145]. The choice of the resection type and extent depends on the lesion location, size, and number and the overall liver function. [146]

A partial hepatectomy encompasses segmentectomy and bi-segmentectomy, typically performed for small, localized lesions to preserve healthy liver tissue. A lobectomy involves the removal of an entire right or left lobe for larger lesions confined to one lobe. An extended lobectomy (hepatectomy) removes an entire lobe plus part of the contralateral lobe. A tri-segmentectomy is reserved for larger or multiple lesions. A wedge resection is applied for superficial lesions or when maximal liver preservation is essential [147,148].

As a consequence, an accurate anatomical evaluation has become fundamental in radiology [149,150,151,152,153], increasingly supported by 3D surgical planning tools. In fact, preoperative CT-based imaging provides detailed anatomical and functional information, which is crucial for surgical planning and improving outcomes [15]. CT imaging allows the precise assessment of the lesion size and location, detailed hepatic vasculature, and biliary anatomy—information essential to select the appropriate surgical approach [154,155]. A volumetric CT analysis supports the functional evaluation of the FLR, helping to predict whether the residual liver volume will sustain its postoperative function [156]. For early HCC or select advanced cases, local treatments such as microwave ablation can be considered to delay surgery [157].

Preoperative CT also facilitates parenchymal assessments—identifying areas of steatosis, fibrosis, or cirrhosis—and disease staging, which impacts the surgical planning and prognosis [158,159]. Furthermore, the evaluation of lesion margins ensures an adequate oncological clearance while preserving the liver function, including in uncommon HCC presentations [160,161]. A nomogram combining CT imaging with serum albumin and total bilirubin levels has demonstrated a good predictive performance for PHLF in resectable HCC patients [162].

Radiomics offers further refinement by identifying patients likely to benefit from local treatments due to unresectable tumors or high postoperative risks [163,164,165,166,167,168,169]. The rapid advancement of AI in hepatology has demonstrated potential for enhanced diagnosis, risk stratification, surgical planning, and transplant allocation [170,171,172,173,174]. DL and ML models have been developed to predict PHLF and support clinical decision-making [172,173].

Despite the progress in radiomics and AI use in the prediction and evaluation of PHLF and the FLR, several critical gaps remain. A first limitation is that the definitions for PHLF across studies are not unique, which complicates comparison and reproducibility. Moreover, the significant heterogeneity in patient populations, imaging protocols, and segmentation methods reduces the standardization of models, which are basically developed on small, single-center cohorts and lack external validation. Even though many studies report high AUCs and a promising predictive accuracy, there is a risk of overfitting, especially when methodological elements are not clearly addressed [175,176].

While this review does not formally apply TRIPOD (Transparent Reporting of a Multivariable Prediction Model for Individual Prognosis Or Diagnosis) or PROBAST (Prediction Model Risk of Bias Assessment Tool) frameworks, these tools actually represent essential standards of quality evaluations in clinical research, to address a more robust endpoint. These guidelines should systematically be a part of future investigations to improve the validation, methodological transparency, and hopefully clinical applicability to reach real-world clinical utility into hepatobiliary surgery.

Wang et al. [177] conducted a rigorous systematic review of 34 prognostic models for PHLF, applying PROBAST criteria and highlighting the high risk of bias and limited generalizability of most current tools. 

A recent narrative review by Mian et al. [178] emphasized the clinical integration of radiomics in liver cancer workflows, focusing on how such tools can inform surgical decision-making and patient stratification. 

Maino et al. [178] provided an overview of radiomics in liver imaging, critically addressing reproducibility, standardization, and translational readiness. These works offer valuable insights from methodological, clinical, and strategic perspectives. In contrast, this review contributes a more focused update on CT-based approaches for FLR and PHLF predictions, highlighting recent AI applications while acknowledging the outstanding limitations that still hinder clinical adoption.

While radiomics holds great potential to enhance preoperative risk stratification in liver surgery, this review highlights that the field is still in its early stages. The lack of multicenter validation, the variability in imaging acquisition and segmentation protocols, and the insufficient transparency in the model development represent significant barriers to clinical translation. Moreover, the interpretability of radiomic features and their integration with clinical variables remain underexplored. These aspects must be addressed through standardized pipelines, large-scale collaborative studies, and an adherence to methodological guidelines to ensure reproducibility and trust in AI-based tools.

## 5. Conclusions

Both PHLF and FLR radiomics models have shown encouraging potential for the preoperative prediction of the hepatic function and residual liver volume. In particular, studies incorporating both clinical and imaging-based features have achieved a high predictive accuracy. However, the overall methodological quality remains heterogeneous. Many models rely on small, single-center datasets, lack external validation, and do not adhere to standardized segmentation protocols. Moreover, few studies assess model interpretability or provide access to the data and source code, limiting reproducibility. These limitations currently hinder the clinical translation of radiomics tools in hepatobiliary surgery. To move forward, future research should prioritize prospective, multicenter validation; transparent reporting; and the integration of radiomics with established clinical predictors. The adherence to methodological frameworks such as TRIPOD, the PROBAST, and the CLEAR will be essential to ensure the robustness, generalizability, and clinical relevance of AI-driven prediction models in this field.

## Figures and Tables

**Table 1:** Summary of radiomics studies for FLR and PHLF prediction and CLEAR criteria consideration.

Study	Prediction Target	Model Type	Sample Size	Validation	Key CLEAR Aspects Considered
Zhu et al. (2023) [86]	FLR function (ICG-R15)	ML, Radiomics	190	Internal	Segmentation clearly reported; internal validation; no external test set; reproducibility not addressed.
Xie et al. (2023) [87]	FLR% volumetry (blood-free)	DL (3D U-Net)	170	External	Well-described segmentation: external test set included metrics reported; lack of data/model sharing.
Xie et al. (2024) [88]	FLR (blood-filled vs. blood-free)	DL (3D U-Net)	178	External	Clear comparison between automated and manual FLR; reproducibility analyzed; performance robust.
Xu et al. (2023) [89]	PHLF	DL	265	Not defined	DL architecture described; lack of validation strategy reduces generalizability; input imaging well defined.
Cai et al. (2019) [90]	PHLF	Radiomics Nomogram	112 (+13 prospective)	Internal + pilot external	Integrated Rad score + MELD + PS; training/test split reported; clinical applicability discussed.
Kang et al. (2024) [20]	PHLF	ML	52	Not defined	Custom loss function: performance evaluated but external validation missing; segmentation unclear.
Xiang et al. (2021) [24]	PHLF (large HCC)	Radiomics Nomogram	186 (131 train/55 test)	internal	Training/test division; model performance reported; segmentation not standardized; reproducibility not assessed.

## Abbreviations

The following abbreviations are used in this manuscript:

FRL	future remnant liver
PHLF	post-hepatectomy liver failure
VIPP	volume-associated ICG-PLT-PT
ICG	indocyanine green clearance test
PLT	platelet count
PT	prothrombin time
ALB	albumin
TB	total bilirubin
